# Comparative Characterization of Vaginal and Gut Microbiota in Late-Pregnancy Women with or Without Group B Streptococcus Colonization

**DOI:** 10.3390/microorganisms13122671

**Published:** 2025-11-24

**Authors:** Shuang Song, Kaori Iino, Mako Nakamura, Maki Sato, Maika Oishi, Asami Ito, Yoshihito Yokoyama

**Affiliations:** Department of Obstetrics and Gynecology, Hirosaki University Graduate School of Medicine, 5 Zaifu, Hirosaki 036-8562, Aomori, Japan; ssong@cmu.edu.cn (S.S.); yokoyama@hirosaki-u.ac.jp (Y.Y.)

**Keywords:** Group B Streptococcus, preterm birth, chorioamnionitis, 16S rRNA sequencing, *Lactobacillus iners*, *Lactobacillus crispatus*, bacterial vaginosis-associated bacteria, microbiome diversity

## Abstract

Group B Streptococcus (GBS) colonization during pregnancy is a major cause of neonatal infection, yet its microbial determinants remain unclear. This pilot study compared the vaginal and gut microbiota of late-pregnancy women with and without GBS colonization to explore potential microbial cues for peripartum risk stratification. Forty-three Japanese pregnant women (GBS-Negative = 34; GBS-Positive = 9) were enrolled at 35–37 weeks of gestation. Vaginal secretions and stool were analyzed by 16S rRNA (V3–V4) sequencing using QIIME 2 with SILVA annotation and community state type (CST) classification. Vaginal communities were mainly Lactobacillus-dominant. GBS-Positive women showed a non-significant tendency toward more *L. iners*-dominant CST III and fewer *L. crispatus*-dominant CST I compared with GBS-Negative women. Prevotella, Atopobium, and Gardnerella were significantly enriched in the GBS-Positive group (false discovery rate < 0.05), whereas gut microbial diversity and composition showed no significant differences between groups. Cross-site gut–vagina genus-level correlations were generally weak and non-significant. These findings suggest that, in late pregnancy, GBS colonization is linked to subtle shifts within Lactobacillus-dominant vaginal communities, with more *L. iners* and bacterial vaginosis-associated genera, rather than global microbiota disruption. The apparent shift from *L. crispatus*- to *L. iners*-dominant communities is hypothesis-generating and should be confirmed in larger cohorts.

## 1. Introduction

*Streptococcus agalactiae* (Group B Streptococcus; GBS) is a Gram-positive bacterium that frequently colonizes the lower gastrointestinal and female genitourinary tracts of healthy adults [1]. Although GBS colonization is typically asymptomatic, it has important clinical implications in obstetrics, being associated with preterm labor, premature rupture of membranes, and neonatal sepsis or meningitis through vertical transmission during childbirth [2,3]. To mitigate these risks, many countries have implemented universal antenatal GBS screening and intrapartum antibiotic prophylaxis, following national obstetric guidelines. The American College of Obstetricians and Gynecologists recommends culture-based screening at 36–37 weeks of gestation [4], whereas the Japan Society of Obstetrics and Gynecology recommends screening between 35 and 37 weeks to best reflect colonization status at delivery [5]. Despite these preventive strategies, the biological mechanisms underlying GBS colonization and host-microbiota interactions remain poorly understood [2].

Recent advances in microbiome research have provided new insights into host–pathogen ecology [6]. The vaginal microbiota (VMB) plays a critical role in reproductive health and pregnancy maintenance [7]. It can be classified into five community state types (CSTs), where Lactobacillus-dominant CSTs (I-III) are generally associated with vaginal health, and CST IV, characterized by low abundance of Lactobacillus and high diversity of anaerobic, bacterial vaginosis (BV)-associated taxa, is linked to dysbiosis and adverse obstetric outcomes [8]. In line with this, a recent cervicovaginal microbiome study comparing early miscarriages with ongoing pregnancies reported that miscarriage cases were characterized by reduced Lactobacillus dominance and enrichment of BV-associated taxa, further underscoring the potential impact of early deviations from Lactobacillus-dominant communities on pregnancy maintenance [9]. Meanwhile, the gut microbiota influences host immunity and metabolism and may indirectly affect vaginal colonization by opportunistic pathogens such as GBS [10]. Emerging evidence supports a bidirectional “gut–vagina axis,” mediated by metabolites (e.g., bile acids, short-chain fatty acids), immune signaling (e.g., IgA, Treg cells), and endocrine factors [11,12,13,14]. However, the specific relationships between GBS colonization and concurrent vaginal and gut microbiota profiles during late pregnancy remain unclear.

Notably, recent work has begun to dissect direct ecological interactions between GBS and BV-associated bacteria, particularly *Gardnerella vaginalis*. In a pregnant mouse model, vaginal exposure to *G. vaginalis* markedly increased the risk of GBS vaginal colonization and ascending uteroplacental infection, whereas inoculation with GBS alone at comparable doses failed to produce sustained colonization or upper tract infection [15]. Complementary in vitro co-culture studies have shown that GBS can promote *G. vaginalis* biofilm formation via the *luxS*/AI-2 quorum-sensing pathway, enhancing biofilm maturation and potentially contributing to BV recurrence [16]. Together, these data support a bidirectional model in which BV-associated dysbiosis facilitates GBS persistence, while GBS in turn stabilizes *G. vaginalis*-dominated biofilms, reinforcing a BV-like ecological niche.

However, most mechanistic and clinical studies to date have focused on women with clinically overt or recurrent BV, or on animal and in vitro models. It therefore remains unclear whether, in pregnant women without diagnosed BV or vaginitis, more subtle or subclinical shifts toward BV-associated taxa might already be present in late pregnancy and associated with an increased risk of rectovaginal GBS colonization. This question is clinically relevant because routine antenatal GBS screening is typically performed in late gestation, and many GBS-Positive women do not meet diagnostic criteria for BV or other forms of vaginitis.

Against this background, we focused on late pregnancy and enrolled women without clinically diagnosed BV or vaginitis in order to minimize confounding by overt vaginal infection. In this setting, rectovaginal GBS colonization may reflect more subtle ecological shifts rather than frank infectious dysbiosis. We therefore performed 16S rRNA gene sequencing of paired vaginal and stool samples obtained from Japanese pregnant women at 35–37 weeks of gestation. We compared microbial diversity and community structure between GBS-Positive and GBS-Negative women, classified the vaginal microbiota into CSTs, and evaluated cross-site (gut–vagina) genus-level correlations. By integrating these analyses, this pilot study aims to characterize vaginal and gut microbiota profiles associated with GBS colonization in late pregnancy and to provide microbiological insights that may inform future mechanistic work and strategies for peripartum risk stratification and prevention.

## 2. Materials and Methods

### 2.1. Study Population and Sample Collection

This study was conducted at Hirosaki University Hospital, Japan, beginning in May 2023. The study population consisted of pregnant women receiving routine antenatal care at the hospital. This study was approved by the Ethics Committee of the Graduate School of Medicine, Hirosaki University, and was conducted in accordance with the 1964 Declaration of Helsinki and its later amendments or comparable ethical standards. Written informed consent for participation and publication of data was obtained from all participants at the time of enrollment.

### 2.2. Study Population and Inclusion/Exclusion Criteria

Inclusion criteria were pregnant women aged ≥18 years, of Japanese nationality, who were receiving routine antenatal care at Hirosaki University Hospital, planning to deliver at the same hospital, and providing written informed consent. Exclusion criteria included: (1) age <18 years; (2) HIV infection; (3) use of systemic antibiotics within 1 month prior to sampling; (4) use of vaginal douches or local treatments (e.g., antibiotics, corticosteroids, or hormonal preparations) within 2 weeks prior to sampling; (5) presence of uncontrolled chronic diseases before pregnancy (e.g., diabetes mellitus, autoimmune diseases, malignancy); (6) drug abuse or heavy smoking (>15 cigarettes/day); (7) laboratory-confirmed sexually transmitted infections (STIs), including *Chlamydia trachomatis*, *Neisseria gonorrhoeae*, *Trichomonas vaginalis*, and *Mycoplasma genitalium*; (8) diagnosis of aerobic vaginitis (AV) or symptomatic vulvovaginal candidiasis (VVC); (9) diagnosis of bacterial vaginosis (BV); (10) use of proton pump inhibitors (PPIs); (11) use of probiotic preparations (oral or vaginal) within 1 month prior to sampling; (12) bowel preparation or colonoscopy within 1 month prior to sampling; (13) fecal microbiota transplantation or immunosuppressive therapy within 3 months prior to sampling; (14) acute urinary tract infection, gastrointestinal infection, or other active bacterial/viral infection during pregnancy; and (15) vaginal douching, pelvic procedures, or sexual intercourse within 48 h prior to sampling, to avoid short-term interference with the microbiota.

In this study, bacterial vaginosis was diagnosed using the Nugent scoring system on Gram-stained vaginal smears. A Nugent score of 7–10 was considered diagnostic of BV, and women with scores ≥7 were excluded from the present analysis.

### 2.3. Sample Collection and Microbiome/GBS Analyses

All participants underwent obstetric evaluation and sample collection at 35–37 weeks of gestation. At this visit, a combined vaginal-rectal swab was obtained for Group B Streptococcus (GBS) culture and screening, and additional vaginal secretions were collected for microbiome analysis. Stool samples for microbiome analysis were self-collected by the participants within the same gestational window (35–37 weeks) using the provided collection kit and returned to the hospital. All microbiome samples were collected into stabilization buffers provided in the commercial kits and were kept at ambient temperature according to the manufacturers’ instructions until they reached the laboratory. Upon receipt, microbial DNA was extracted as soon as possible, and the resulting DNA extracts were stored at −80 °C until further analysis.

Clinical data were collected through standardized interviews and medical records, including maternal age, gestational age at sampling, gravidity, parity, mode of conception (spontaneous or infertility treatment), pregnancy complications (e.g., gestational diabetes mellitus, hypertensive disorders of pregnancy, thyroid dysfunction), pregnancy outcomes (mode of delivery, gestational age at birth, neonatal birth weight), maternal body mass index (BMI) in early pregnancy and before delivery, and neonatal outcomes (birth weight, neonatal asphyxia, Apgar scores at 1 and 5 min). Urogenital symptoms were also recorded. Infertility treatment included ovulation induction with timed intercourse, intrauterine insemination (AIH), and assisted reproductive technologies (ART), such as IVF/ICSI and frozen embryo transfer (FET).

DNA extraction and sequencing: Vaginal secretions were collected using the OMNIgene^®^ Vaginal swab kit (OMR-130, DNA Genotek, Ottawa, ON, Canada), and stool samples were obtained with the FS-0017 stool collection kit (TechnoSuruga Laboratory Co., Ltd., Shizuoka, Japan), following the manufacturers’ instructions. Microbial DNA was extracted using the QIAamp PowerFecal Pro DNA Kit (QIAGEN, Hilden, Germany) and quantified with NanoDrop and Qubit assays. The V3-V4 hypervariable regions of the 16S rRNA gene were amplified with a two-step PCR approach. Sequencing libraries were prepared and subjected to paired-end sequencing (2 × 300 bp) on an Illumina MiSeq or NextSeq platform. Library preparation and sequencing were performed by Bioengineering Lab. Co., Ltd. (Sagamihara, Kanagawa, Japan).

GBS culture and screening: Combined vaginal-rectal swabs were processed using a Group B Streptococcus (GBS) selective enrichment and culture system (Eiken Chemical Co., Ltd., Tokyo, Japan) in accordance with Japanese obstetric guidelines and the manufacturer’s instructions. Swabs were first inoculated into a GBS selective enrichment broth and incubated at 35–37 °C for 18–24 h, then subcultured onto a chromogenic GBS agar and 5% sheep blood agar for a further 18–24 h. Colonies suggestive of GBS were confirmed by Gram staining (Gram-positive cocci in chains), catalase testing (negative), and CAMP testing (positive), with additional confirmation by an immunochromatographic rapid antigen assay (Rapid Test Strep B^®^, Eiken Chemical Co., Ltd., Tokyo, Japan) when available. Women were classified as GBS-Positive if *Streptococcus agalactiae* was identified from the combined vaginal-rectal swab by selective culture with at least one positive confirmatory test. Separate vaginal or rectal cultures were not performed; therefore, the GBS status used in all analyses reflects guideline-based late-pregnancy vaginal-rectal colonization and was treated as a binary variable (GBS-Positive vs. GBS-Negative).

### 2.4. Statistical Analysis of Baseline Characteristics

Epidemiological data were analyzed using R (version 4.4.0, 2024-04-24; R Foundation for Statistical Computing, Vienna, Austria). For categorical variables, Fisher’s exact test was applied due to the small sample size and the unequal group distribution (GBS-Positive *n* = 9 vs. GBS-Negative *n* = 34). For continuous variables, Student’s t-test was used when the data met assumptions of normality and homogeneity of variance; otherwise, the Mann–Whitney U test was performed. A two-sided *p*-value < 0.05 was considered statistically significant.

### 2.5. Microbiota Data Analysis

#### 2.5.1. Sequencing and Preprocessing

Vaginal secretion samples and stool samples, collected separately as described above, were subjected to 16S rRNA gene sequencing of the V3-V4 region on the Illumina MiSeq platform (Illumina, San Diego, CA, USA). Raw paired-end reads were demultiplexed and imported into QIIME 2 (version 2024.10) [17]. Per-base quality plots were inspected, and low-quality bases were removed using the q2-dada2 denoise-paired workflow, trimming primer sequences and truncating reads to retain regions with Phred quality scores ≥20. DADA2 (version 2024.10) performed error-model-based denoising, paired-end read merging, chimera removal, and inference of amplicon sequence variants (ASVs), yielding feature tables and representative sequences for downstream analyses.

Taxonomic assignment was performed against the SILVA reference database (release 138) [18]. Vaginal microbiota were analyzed at both genus and species levels; Lactobacillus ASVs were additionally compared against the DDBJ database [19] to refine species-level classification and to assign samples to community state types (CSTs) according to established criteria, in which the vaginal microbiota are categorized into five major groups: CST I (dominated by *L*. *crispatus*), CST II (*L. gasseri*), CST III (*L. iners*), CST IV (characterized by low abundance of Lactobacillus and high diversity of anaerobic bacteria, often associated with bacterial vaginosis), and CST V (*L. jensenii*) [8]. Gut microbiota, in contrast, were summarized at the genus level for downstream analyses.

#### 2.5.2. Microbiota Community Composition, Diversity, and Visualization

Community composition. ASV feature tables and taxonomic annotations generated in QIIME 2 were exported for downstream processing in R. ASV counts were taxonomically collapsed to the genus level and, for the vaginal microbiota, additionally to the species level where available. For each sample, counts were summed within taxa and converted to relative abundances by dividing by the total sequence counts per sample. For the vaginal microbiota, the top four genera and top nine species (ranked by mean relative abundance across all participants) were visualized as stacked bar plots stratified by GBS status; for the gut microbiota, the top twenty-five genera were displayed in the same manner. In all plots, remaining low-abundance taxa were aggregated as “Others.” Data wrangling and visualization were performed in R, and stacked bar plots were generated with ggplot2.

Alpha-diversity (Shannon). Per-sample Shannon diversity (H′) was computed in QIIME 2 using the q2-diversity plugin (alpha, metric = shannon, natural-log base). The resulting Shannon entropy values were exported and imported into R for visualization (boxplots with jittered points overlaid) and statistical analysis. Between-group differences in Shannon diversity (GBS-Positive vs. GBS-Negative) were assessed using two-sided Wilcoxon rank-sum tests. Effect sizes were quantified with Cliff’s delta (δ) and 95% confidence intervals. When multiple pairwise comparisons were performed in other analyses, *p* values were adjusted for multiple testing using the Benjamini–Hochberg false discovery rate (FDR) procedure.

Beta-diversity and ordination. Bray–Curtis dissimilarities were computed in QIIME 2 using the q2-diversity plugin (beta, metric braycurtis), followed by principal coordinate analysis (PCoA) using the QIIME 2 pcoa action. The resulting distance matrix and PCoA coordinates were exported to R for downstream statistical testing and visualization. Between-group differences in overall community composition were evaluated using PERMANOVA (vegan: adonis2, 999 permutations), and ordination plots were generated with the vegan and ggplot2 packages (version 4.0.0).

#### 2.5.3. Differential Abundance and Association Analysis

Associations between genus-level relative abundances and GBS colonization status were examined using the R package MaAsLin2 (version 1.20.0) [20]. Features present in fewer than 10% of samples were excluded to minimize the influence of rare taxa. Data were normalized by total sum scaling (TSS) and log-transformed (LOG) to account for compositionality. GBS colonization status (positive vs. negative) was included as the sole fixed effect. Genera were considered significantly different between groups if they remained significant after false discovery rate (FDR) correction with q < 0.05. The sign of the MaAsLin2 coefficient indicates directionality (positive, enriched in the GBS-Positive group; negative, enriched in the GBS-Negative group; reference = GBS-Negative). Where indicated, we also report features meeting a prespecified exploratory threshold (q < 0.25), which are clearly labeled as exploratory and interpreted cautiously.

#### 2.5.4. Vaginal-Gut Microbiota Correlation Analysis

Gut–vaginal correlation analysis (genus level). Genus-level relative abundance tables exported from QIIME 2 were paired by participant ID (gut vs. vaginal); only participants with both habitats were retained, and analyses focused on the 23 genera shared between tables. To address compositionality, we applied a centered log-ratio (CLR) transform within each habitat on the full genus table per sample (pseudocount 1 × 10^−6^, row closure to 1, natural-log base), then subset to the 23 shared genera. For each genus, gut–vaginal Spearman correlations (two-sided) were computed across paired subjects, with Benjamini–Hochberg adjustment and q-values reported (q < 0.05 significant). We repeated the workflow stratified by GBS status (Negative vs. Positive). Results were visualized as heatmaps of genus-wise Spearman ρ; all analyses were performed in R (tidyverse, compositions, pheatmap/ggplot2).

## 3. Results

### 3.1. Maternal Baseline Characteristics and Delivery Outcomes Across GBS Status Groups

A total of 43 pregnant women were included, of whom 34 were GBS-Negative and 9 were GBS-Positive. Table 1 summarizes the maternal baseline characteristics and delivery-related parameters across the three groups. No statistically significant differences were observed between groups in maternal baseline characteristics or delivery outcomes (all *p* > 0.05). Specifically, maternal age (median [IQR]) was 37.00 [33.00–38.75] years in the GBS-Negative group and 30.00 [29.00–38.00] years in the GBS-Positive group (*p* = 0.192). Pre-pregnancy BMI (kg/m^2^) was 21.60 [19.37–26.80] vs. 21.99 [19.52–24.65] (*p* = 0.846), and BMI (kg/m^2^) at delivery was 26.55 ± 4.37 vs. 27.34 ± 3.59 (*p* = 0.586). Gestational age at delivery (weeks) was identical between groups (39.00 [38.00–40.00] vs. 39.00 [38.00–40.00]; *p* = 0.725). Infant birthweight (g) was 2998.85 ± 404.00 vs. 3194.11 ± 328.95 (*p* = 0.153). For categorical outcomes, the proportion of multiparous women was 61.8% vs. 55.6% (*p* = 1.000); the proportion undergoing infertility treatment was 55.9% vs. 44.4% (*p* = 0.711); and the rate of vaginal delivery was 76.5% vs. 77.8% (*p* = 1.000). No adverse neonatal outcomes were observed in either group. Collectively, these data indicate no significant differences between GBS-Negative and GBS-Positive groups in the assessed baseline characteristics or delivery outcomes.

### 3.2. Vaginal Microbiota

#### 3.2.1. CST Composition

**Figure 1:**
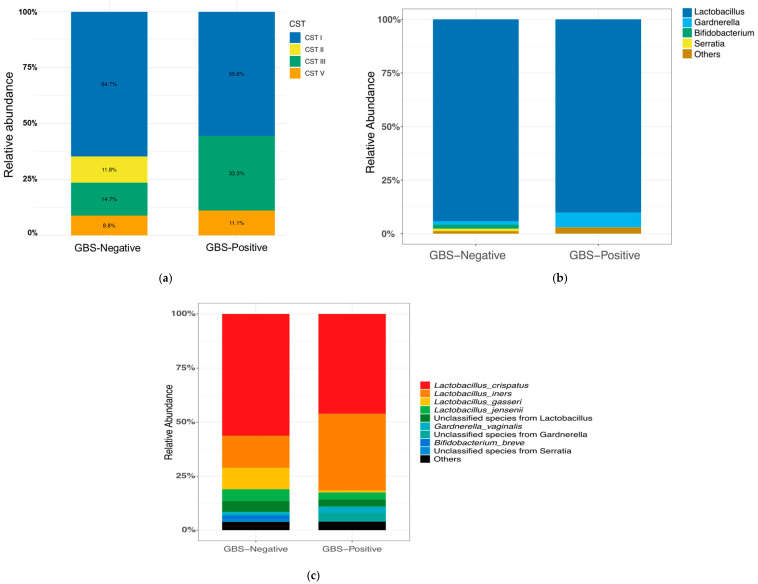

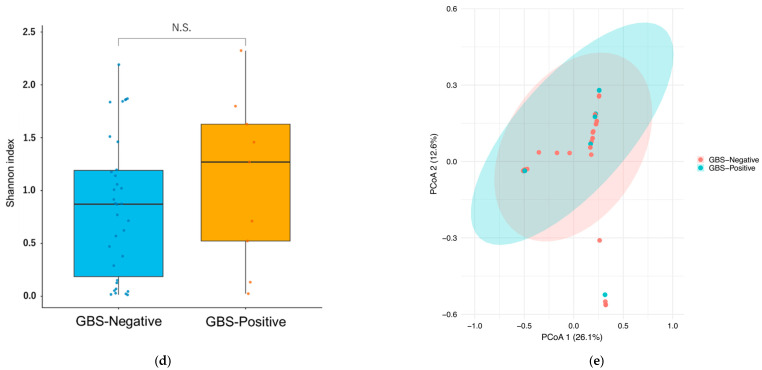
**Vaginal CST distribution, composition, and diversity by GBS colonization status.** (**a**) Distribution of community state types (CST I–V). CST I (*L. crispatus*-dominant) predominated in the GBS-Negative group, whereas the GBS-Positive group showed a higher proportion of CST III (*L. iners*-dominant). No CST IV (anaerobe-dominant) was observed. (**b**) Genus-level composition (top four taxa; remaining taxa aggregated as “Others”). (**c**) Species-level composition (top nine taxa; remaining taxa aggregated as “Others”). (**d**) Shannon diversity (α-diversity) by GBS status. Boxes show the median and interquartile range, whiskers denote the range, and colored circles indicate individual samples.No significant difference was observed between groups (two-sided Wilcoxon rank-sum test, *p* = 0.45; Cliff’s δ = −0.17). (**e**) Bray–Curtis PCoA (β-diversity) by GBS status showing overlapping community structures (PERMANOVA, 999 permutations, *p* = 0.68). Points are colored by group; ellipses show 95% confidence intervals. Abbreviations: CST, community state type; GBS, Group B Streptococcus; PCoA, principal coordinate analysis; PERMANOVA, permutational multivariate analysis of variance.

Based on Lactobacillus-dominance patterns, the vaginal microbiota was classified into community state types (CSTs: I = *L. crispatus*-dominant; II = *L. gasseri*-dominant; III = *L. iners*-dominant; IV = non-Lactobacillus-dominant/high-diversity; V = *L. jensenii*-dominant). In our study, the GBS-Negative group (*n* = 34) was predominantly CST I (22/34, 64.7%), followed by CST III (5/34, 14.7%), CST II (4/34, 11.8%), and CST V (3/34, 8.8%). The GBS-Positive group (*n* = 9) was likewise dominated by CST I (5/9, 55.6%) but showed a higher proportion of CST III (3/9, 33.3%); CST V accounted for 1/9 (11.1%), whereas CST II was not observed (0/9). No CST IV was detected in either group. An R × C Fisher’s exact test showed no significant difference in overall CST composition between GBS-Positive and GBS-Negative women (*p* = 0.606). Pairwise Fisher’s exact tests for each CST category were also non-significant after Benjamini–Hochberg correction (all q > 0.05). Descriptively, CST III appeared more frequent in the GBS-Positive group, whereas CST II was only observed in GBS-Negative women (Figure 1a); however, these patterns should be interpreted cautiously given the small sample size.

#### 3.2.2. Vaginal Microbial Community Composition

In the QIIME 2 analysis, sequences assigned to Archaea, Eukaryota, Chloroplast, and Mitochondria were excluded to ensure accurate taxonomic summarization. Across vaginal samples, 36 genus-level taxa and 64 species-level taxa (where resolvable) showed non-zero relative abundance. At the genus level, the GBS-Negative group (*n* = 34) was dominated by Lactobacillus (94.23%), followed by Bifidobacterium (1.84%); together these top genera comprised 96.07% of the community. The GBS-Positive group (*n* = 9) was likewise dominated by Lactobacillus (90.13%), with Gardnerella (6.96%) as the next most abundant genus; these two genera accounted for 97.09% of the community. Figure 1b compares the top four taxa (by mean relative abundance) between groups.

At the species level, the most abundant taxa in the GBS-Negative group were *L. crispatus* (56.32%), *L*. *iners* (14.88%), *L. gasseri* (9.85%), and *L. jensenii* (5.42%). In the GBS-Positive group, the leading taxa were *L. crispatus* (46.18%), *L. iners* (35.52%), *Gardnerella* sp. (4.21%, not resolved to species), and *L. jensenii* (3.31%). Figure 1c summarizes the top 9 taxa at the species level.

#### 3.2.3. Vaginal Microbiota Diversity

Vaginal α-diversity (Shannon index). In the comparison between GBS-Negative (*n =* 34) and GBS-Positive (*n* = 9) participants, vaginal Shannon diversity values were generally modest in both groups, consistent with communities dominated by one or a few Lactobacillus species (Figure 1d). The distribution of Shannon indices showed considerable overlap, with a slightly higher median in the GBS-Positive group but substantial inter-individual variability in both groups. This difference was not statistically significant (two-sided Wilcoxon rank-sum test, *p* = 0.45). Cliff’s delta (δ; GBS-Negative vs. GBS-Positive) was −0.17, indicating only a small, non-significant effect size, compatible with at most a weak tendency toward higher α-diversity among GBS-Positive women. Overall, we did not detect a clear difference in vaginal Shannon diversity according to GBS colonization status.

Vaginal β-diversity (Bray–Curtis, PCoA). Principal coordinate analysis (PCoA) based on Bray–Curtis dissimilarities likewise showed no obvious separation between GBS-Negative and GBS-Positive vaginal samples (Figure 1e). Points from the two groups were largely intermingled in ordination space, and the 95% confidence ellipses for each group overlapped extensively. Consistent with this visual impression, PERMANOVA on the Bray–Curtis distance matrix did not identify a significant difference in overall community composition between groups (999 permutations, *p* = 0.68). The spread of points suggested comparable within-group dispersion as well. Taken together, these findings provide no evidence that vaginal β-diversity differs meaningfully between GBS-Negative and GBS-Positive women in this study.

### 3.3. Gut Microbiota

#### 3.3.1. Gut Microbial Community Composition

**Figure 2:**
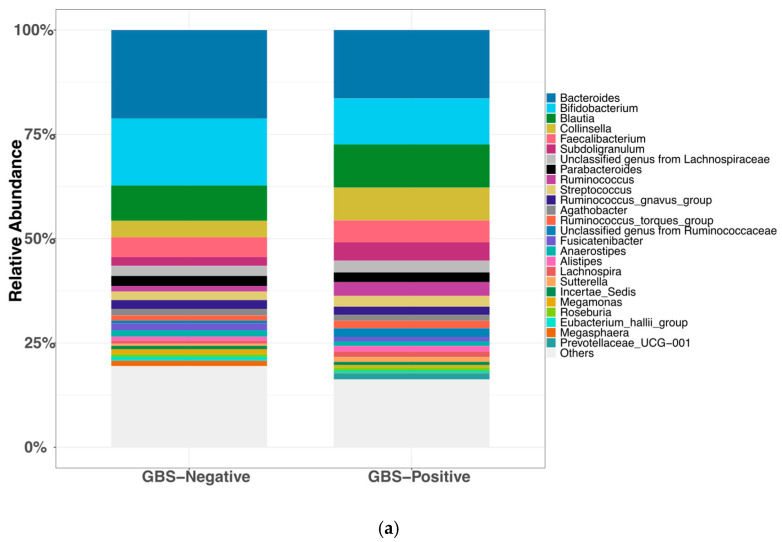

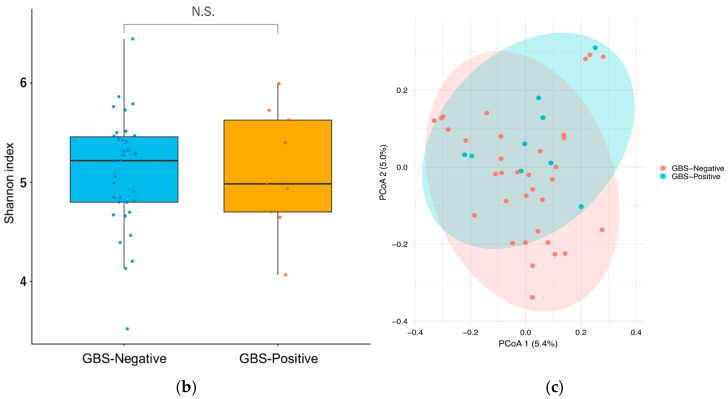
**Gut microbiota composition and diversity by GBS colonization status.** (**a**) Relative abundance of the top 25 genus-level taxa by group (ranked by mean across all samples; remaining taxa aggregated as “Others”). (**b**) Shannon diversity (α-diversity) by GBS status. Boxes show the median and interquartile range, whiskers denote the range, and colored circles indicate individual samples.no significant difference (two-sided Wilcoxon rank-sum test, *p* = 0.96; Cliff’s δ = −0.01). (**c**) Bray–Curtis PCoA of gut communities (β-diversity) by GBS status (points colored by group; 95% confidence ellipses); group difference not significant (PERMANOVA, 999 permutations, *p* = 0.11). Abbreviations: PCoA, principal coordinate analysis; PERMANOVA, permutational multivariate analysis of variance; GBS, Group B Streptococcus.

Gut microbiota was processed identically and summarized at the lowest confidently assigned rank (genus when resolvable). In total, 206 taxa were detected at non-zero relative abundance across stool samples. In the GBS-Negative group (*n* = 34), the gut microbiota was dominated by Bacteroides (21.21%), Bifidobacterium (16.04%), Blautia (8.44%), Faecalibacterium (4.72%), and Collinsella (3.98%), accounting for 54.41% of the overall community. Similarly, in the GBS-Positive group (*n* = 9), the predominant genera were Bacteroides (16.39%), Bifidobacterium (11.02%), Blautia (10.31%), Collinsella (7.90%), and Faecalibacterium (5.21%), which together comprised 50.86% of the community. Figure 2a summarizes the relative abundance of the top 25 taxa across groups.

#### 3.3.2. Diversity Analysis of Gut Microbiota

Gut α-diversity (Shannon index). Gut Shannon diversity was slightly higher in the GBS-Positive group (*n* = 9) than in the GBS-Negative group (*n* = 34), but this difference was not statistically significant (two-sided Wilcoxon rank-sum test, *p* = 0.96) (Figure 2b). Cliff’s delta (δ) (GBS-Negative vs. GBS-Positive) was −0.01, indicating a small, non-significant effect size and no clear difference in gut α-diversity between groups.

Gut β-diversity (Bray–Curtis, PCoA). Principal coordinate analysis (PCoA) based on Bray–Curtis dissimilarities showed substantial overlap between GBS-Negative and GBS-Positive samples, without obvious group separation (Figure 2c). Consistent with this, PERMANOVA on the Bray–Curtis distance matrix did not detect a significant difference in overall gut community composition between groups (999 permutations, *p* = 0.11). Taken together, these results suggest that neither gut α- nor β-diversity differed appreciably by GBS colonization status in this study.

### 3.4. Differential Abundance of Vaginal and Gut Microbiota

**Figure 3:**
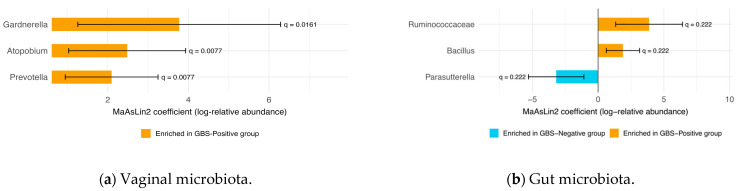
**Differentially abundant taxa by GBS colonization status (MaAsLin2).** (**a**) Vaginal microbiota: three taxa remained significant after FDR correction (q < 0.05)—Prevotella, Atopobium, and Gardnerella—all enriched in the GBS-Positive group. Bars show MaAsLin2 coefficients (log-relative abundance) with 95% confidence intervals; q-values are annotated at the ends of the bars. (**b**) Gut microbiota: taxa meeting an exploratory threshold (q < 0.25) are shown—Bacillus and family Ruminococcaceae enriched in the GBS-Positive group, and Parasutterella enriched in the GBS-Negative group. Bars represent MaAsLin2 coefficients (log-relative abundance) with 95% confidence intervals; q-values are shown at bar ends. (Taxonomic ranks are indicated where not at genus level.) Abbreviations: FDR, false discovery rate; GBS, Group B Streptococcus.

Using MaAsLin2, we examined genus-level differential abundance in relation to GBS colonization for both the vaginal and gut microbiota. In the vaginal microbiota, three genera remained statistically significant after FDR correction (q < 0.05). Prevotella (coefficient = 2.10, standard error = 0.59, *p* = 0.0009, q = 0.0077), Atopobium (coefficient = 2.48, standard error = 0.74, *p* = 0.0018, q = 0.0077), and Gardnerella (coefficient = 3.77, standard error = 1.29, *p* = 0.0054, q = 0.0161) were all enriched in the GBS-Positive group compared with the GBS-Negative group, whereas no other genera showed significant differences (q ≥ 0.05) (Figure 3a).

In the gut microbiota, no taxa reached significance under the stringent FDR threshold of q < 0.05. However, when a more relaxed cutoff of q < 0.25 was applied, several taxa showed associations with GBS colonization status. Bacillus (coefficient = 1.888, standard error = 0.646, *p* = 0.00564, q = 0.22175) and the Ruminococcaceae family (coefficient = 3.878, standard error = 1.296, *p* = 0.00467, q = 0.22175) were enriched in the GBS-Positive group, whereas *Parasutterella* (coefficient = −3.186, standard error = 1.077, *p* = 0.00511, q = 0.22175) was more abundant in the GBS-Negative group (Figure 3b).

### 3.5. Spearman Correlation Analysis of Shared Vaginal and Gut Genera

**Figure 4:**
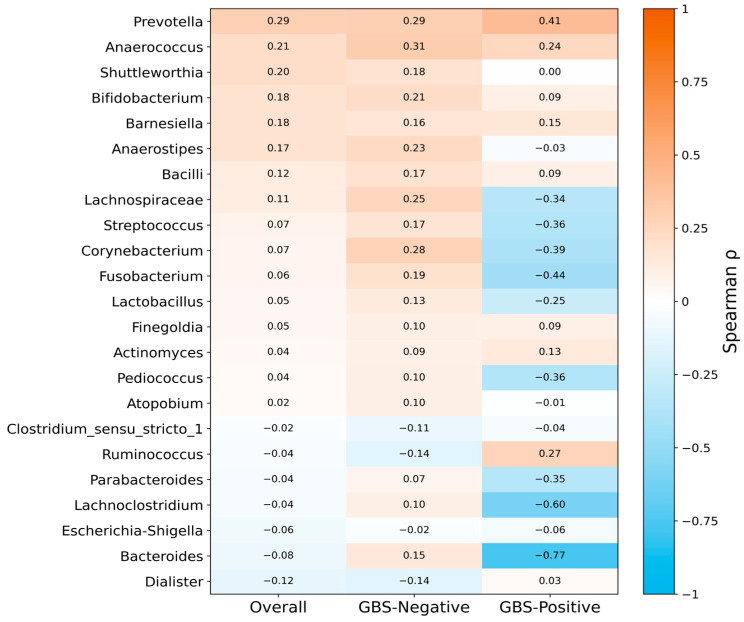
**Genus-level gut–vaginal microbial correlations in late pregnancy, stratified by GBS colonization status.** Heatmap of Spearman correlation coefficients (ρ) for 23 genera shared between vaginal and gut microbiota. Overall correlations were generally weak and mostly positive, and none remained significant after false discovery rate (FDR) correction. Stratified analyses indicated that positive correlations predominated in the GBS-Negative group, whereas correlations tended to be more often negative in the GBS-Positive group, which may be consistent with disrupted gut–vaginal microbial concordance under GBS colonization. Relative abundances were CLR-transformed before correlation analysis. Abbreviations: CLR, centered log-ratio; FDR, false discovery rate; GBS, Group B Streptococcus.

At the genus level, 23 taxa were shared between the vaginal and gut microbiota, and their gut–vaginal associations were assessed using Spearman’s rank correlation (Figure 4). Across all 43 paired samples, correlations between vaginal and gut abundances were generally weak (overall Spearman’s ρ ranged from −0.12 to 0.29; median ρ = 0.05, interquartile range [IQR] 0.03–0.15), and most genera showed positive rather than negative correlations (16/23 genera with ρ > 0). However, none of these associations remained statistically significant after correction for multiple testing (all q ≥ 0.05).

In stratified analyses, distinct patterns emerged by GBS status. In the GBS-Negative group (*n* = 34 pairs), gut–vaginal correlations were modest but predominantly positive (ρ range −0.14 to 0.31; median ρ = 0.15, IQR 0.09–0.20; 19/23 genera with ρ > 0), suggesting a tendency toward concordant shifts in gut and vaginal communities in the absence of colonization. By contrast, in the GBS-Positive group (*n* = 9 pairs), correlations shifted toward negative values (ρ range −0.77 to 0.41; median ρ = −0.03, IQR −0.36–0.09; 13/23 genera with ρ < 0), consistent with a possible transition from predominantly cooperative to more decoupled or even antagonistic gut–vaginal relationships under GBS colonization. Nevertheless, no individual genus-level correlation remained significant after FDR adjustment, which may in part reflect the limited sample size in the GBS-Positive subgroup.

## 4. Discussion

In this late-pregnancy study, rectovaginal GBS colonization was associated with selective compositional features in the vaginal microbiota rather than broad shifts across body sites. At the CST level, the proportion of CST III (*L. iners*-dominant) tended to be higher, and CST I (*L. crispatus*-dominant) tended to be lower in GBS-Positive women; however, these differences did not reach statistical significance. By contrast, at the taxon level, in the vaginal microbiota, MaAsLin2 identified three BV-associated genera-Gardnerella, Atopobium, and Prevotella—significantly enriched in the GBS-Positive group after FDR correction (q < 0.05). In the gut, no genus-level differences met the prespecified significance threshold after multiple-testing correction, and gut–vagina correlations were weak and non-significant in this pilot study. Taken together, these findings indicate that GBS colonization is linked to targeted changes in the vaginal community, while CST shifts and gut compositional differences were not statistically supported.

Previous studies have shown that *L. crispatus*-dominated communities (CST I) are characterized by lower vaginal pH, lower α-diversity, and greater stability, and are therefore often regarded as a relatively optimal state [8,21]. By contrast, *L. iners* has more limited acidogenic capacity, and *L. iners*-dominated communities (CST III) are commonly viewed as suboptimal or transitional, with a greater propensity to shift toward BV-like, high-diversity communities (CST IV) when perturbed [22,23,24,25]. Consistent with this notion, a recent large-scale meta-analysis of vaginal microbiota in women of reproductive age reported that CST I (*L. crispatus*-dominant) was enriched among healthy controls, whereas CST III and CST IV were strongly associated with bacterial vaginosis and other genital infections [26]. In our study (excluding women with clinically diagnosed BV or vaginitis), the proportion of CST III and the relative abundance of *L. iners* were nominally higher in GBS-Positive women, whereas CST I and *L. crispatus* tended to be more prevalent in GBS-Negative women; however, none of these differences reached statistical significance. Nevertheless, the directional pattern aligns with prior evidence: a longitudinal study in pregnancy suggested that persistent GBS colonization is accompanied by decreases in *L. crispatus* with concomitant increases in *L. iners* [27], and a large cross-sectional study (*n* = 1860) reported an inverse association between *L. crispatus* and GBS carriage [3]. Given our limited sample size, these observations should be interpreted as hypothesis-generating rather than definitive; nonetheless, they provide useful clues to the vaginal ecological features associated with GBS in women in late pregnancy without clinical BV, warranting confirmation in larger, longitudinal cohorts.

At the vaginal level, multivariable analysis with MaAsLin2 showed that the relative abundances of Gardnerella, Atopobium, and Prevotella were significantly higher in GBS-Positive women after FDR correction (q < 0.05), indicating a statistical association between these canonical BV-associated genera and GBS colonization. These organisms exhibit traits such as epithelial adherence, biofilm formation/maturation, elevation of vaginal pH, disruption of mucosal barriers, and pro-inflammatory activity; within a biofilm microenvironment they may act synergistically, thereby weakening the *Lactobacillus*-maintained acidic barrier and vaginal homeostasis [28,29,30,31]. Notably, this enrichment was observed despite exclusion of women with clinically diagnosed BV or vaginitis, suggesting that subclinical BV-like shifts may co-occur with GBS colonization. This observation is consistent with external evidence: in a pregnant mouse model, Gardnerella colonization increased the vaginal GBS burden and the risk of ascending uteroplacental infection [15], and clinical data reported a higher detection rate of Gardnerella among GBS-Positive versus GBS-Negative pregnant women (≈23% vs. 4%) [32]. As noted in the Introduction, in vitro co-culture studies indicate that GBS-derived *luxS*/AI-2 quorum-sensing can promote *G. vaginalis* biofilm maturation [16], providing a plausible mechanistic basis for mutual reinforcement. Overall, the observed population-level associations are biologically plausible: BV-associated genera correlate positively with GBS colonization and may form a self-reinforcing network through biofilm cooperation, higher vaginal pH, greater adhesion, and barrier disruption. Future large longitudinal cohorts and functional studies are required to confirm causality and assess microecology-targeted interventions.

By contrast, we detected no significant genus-level differences in the gut microbiota by GBS status after FDR correction. Dominant genera (e.g., Bacteroides, Bifidobacterium, Blautia, Faecalibacterium) showed similar profiles across groups. Using a relaxed, discovery-oriented threshold (q < 0.25), we noted nominal trends toward higher Ruminococcaceae and Bacillus in GBS-Positive stools and higher Parasutterella in GBS-Negative stools; however, these signals did not meet our primary significance criterion and should be considered hypothesis-generating. Given the a priori genus-level focus, limited species-level resolution of V3-V4 amplicons for many gut clades, and the modest sample size, we refrain from further interpretation; these features may warrant targeted evaluation in larger longitudinal cohorts and functional assays.

Similarly, gut–vagina correlation analyses indicated that in late pregnancy, there was no clear synchrony in genus-level composition between the two sites. In both the overall cohort and analyses stratified by GBS status, most genus-level gut–vaginal correlations were weak and did not reach statistical significance. These findings suggest that the so-called “gut–vagina axis” may not primarily manifest as synchronous shifts in taxonomic composition at this stage of gestation. Instead, rising estrogen levels and local immune remodeling in late pregnancy may bias the vaginal niche toward Lactobacillus dominance, creating a relatively compartmentalized vaginal ecological barrier that buffers distal influences from the gut [33].

When we examined patterns by GBS status, stratified analyses nevertheless revealed certain tendencies: gut–vaginal correlations were weakly positive overall in GBS-Negative women, whereas they tended to be more negative in GBS-Positive women. This pattern raises the possibility that GBS colonization and the associated vaginal dysbiosis may attenuate or even disrupt coordination between the two ecosystems. However, these tendencies did not remain statistically significant after correction for multiple testing, likely due to the small number of GBS-Positive cases (*n* = 9) and substantial inter-individual variability. Taken together, our data did not identify clear genus-level coupling between the gut and vaginal microbiota and instead support the notion that gut influences on the vagina may operate mainly through metabolic, immunological, and endocrine pathways rather than through simple taxonomic concordance.

Beyond these cross-sectional observations, this study points to several avenues for future research. First, longitudinal designs are needed to clarify the causal relationship between GBS and the microbiota. Repeated sampling across pregnancy (and postpartum) could help determine whether the transition from *L. crispatus*- to *L. iners*-dominated communities precedes GBS acquisition, or whether GBS itself drives vaginal community restructuring, as well as distinguish transient from persistent carriers [34]. Second, multi-omics and high-resolution approaches will be essential to resolve functional dynamics. Metagenomics can be used to determine strain relatedness (e.g., whether rectal and vaginal GBS isolates are clonal and to trace transmission pathways) and to characterize genetic potential for biofilm formation or bacteriocin production. Integration of metatranscriptomics, metabolomics, and host immunologic profiling would allow community shifts to be linked to functional readouts such as vaginal pH, mucosal immune status, and levels of metabolites including lactic acid, short-chain fatty acids, and amines.

In this context, our findings also inform potential intervention strategies [35]. Augmenting protective vaginal microbiota through probiotic supplementation represents a promising approach. Several studies have reported that specific probiotic interventions can partially restore protective lactobacilli and reduce GBS colonization or increase GBS clearance during pregnancy [36,37,38]. Combining probiotics and prebiotics with targeted intrapartum antibiotic prophylaxis may not only reduce overall antibiotic use in pregnancy but also help re-establish *L. crispatus*-dominated vaginal communities, thereby mitigating GBS-related risk.

In the longer term, a deeper understanding of the gut–vagina axis and the identification of microbial predictors of GBS colonization will be crucial for advancing personalized obstetric care. The exploratory findings of this pilot study require confirmation in larger and more diverse cohorts, ideally incorporating integrated multi-omics analyses.

## 5. Conclusions

In this study of late-pregnant women without clinically diagnosed vaginitis or BV, rectovaginal GBS colonization was not associated with significant differences in vaginal or gut diversity indices or with robust genus-level coupling between the two sites. GBS-Positive women showed a numerically higher frequency of *L. iners*-dominated CST III and a lower prevalence of *L. crispatus*-dominated CST I, although these differences did not reach statistical significance, and they exhibited a significant enrichment of BV-associated genera (Gardnerella, Atopobium, Prevotella) in the vaginal microbiota, whereas no gut taxa remained associated with GBS after multiple-testing correction. These findings suggest that, in late pregnancy, GBS colonization may be linked to selective, BV-like perturbations of the vaginal niche rather than to broad maternal dysbiosis, but they should be interpreted as exploratory; larger, longitudinal, multi-omic studies are needed to confirm these observations and to evaluate microbiota-targeted strategies aimed at preserving or restoring a *L. crispatus*-dominated vaginal community to mitigate GBS-related perinatal risks.

## Figures and Tables

**Table 1:** Maternal baseline characteristics and delivery outcomes across Group B Streptococcus (GBS) Status.

Variable	Overall (*n*= 43)	Negative (*n* = 34)	Positive (*n* = 9)	*p*-Value
Maternal age (years)	36.00 [32.00, 38.50]	37.00 [33.00, 38.75]	30.00 [29.00, 38.00]	0.192 ^a^
Pre-pregnancy BMI (kg/m^2^)	21.63 [19.39, 26.76]	21.60 [19.37, 26.80]	21.99 [19.52, 24.65]	0.846 ^a^
BMI at delivery (kg/m^2^)	26.71 ± 4.19	26.55 ± 4.37	27.34 ± 3.59	0.586 ^b^
Gestational age at delivery (weeks)	39.00 [38.00, 40.00]	39.00 [38.00, 40.00]	39.00 [38.00, 40.00]	0.725 ^a^
Infant Birth weight (g)	3039.72 ± 394.10	2998.85 ± 404.00	3194.11 ± 328.95	0.153 ^b^
Parity, *n* (%)				1 ^c^
—Multiparous/Primiparous	26 (60.5%)	21 (61.8%)	5 (55.6%)	
—Nulliparous	17 (39.5%)	13 (38.2%)	4 (44.4%)	
Infertility Treatment (non-spontaneous conception), *n* (%)				
Yes	23 (53.5%)	19 (55.9%)	4 (44.4%)	0.711 ^c^
No	20 (46.5%)	15 (44.1%)	5 (55.6%)	
Mode of delivery, *n* (%)				
Vaginal	33 (76.7%)	26 (76.5%)	7 (77.8%)	1 ^c^
Cesarean	10 (23.3%)	8 (23.5%)	2 (22.2%)	

^a^ Mann–Whitney U test; ^b^ Welch’s *t* test; ^c^ Fisher’s exact test. Continuous variables are summarized as median [IQR] or mean ± SD as appropriate; categorical variables as *n* (%).

## Data Availability

The raw sequencing data supporting the findings of this study have been deposited in the DNA Data Bank of Japan (DDBJ). The Fastq files of the bacterial microbiota analyzed in this study are available under the BioSample submission ID: SSUB043931. Further inquiries can be directed to the corresponding author.
